# Carotidynia Alias Transient Perivascular Inflammation of the Carotid Artery (TIPIC Syndrome)

**DOI:** 10.5334/jbsr.1595

**Published:** 2018-08-02

**Authors:** Bruno Coulier, Stéphane Van den Broeck, Geoffrey C. Colin

**Affiliations:** 1 Clinique Saint-Luc, Bouge, Namur, BE

**Keywords:** Carotidynia, TIPIC Syndrome, MRI, Doppler ultrasonography, Contrast-enhanced ultrasound

## Case Report

**Figure 1:**
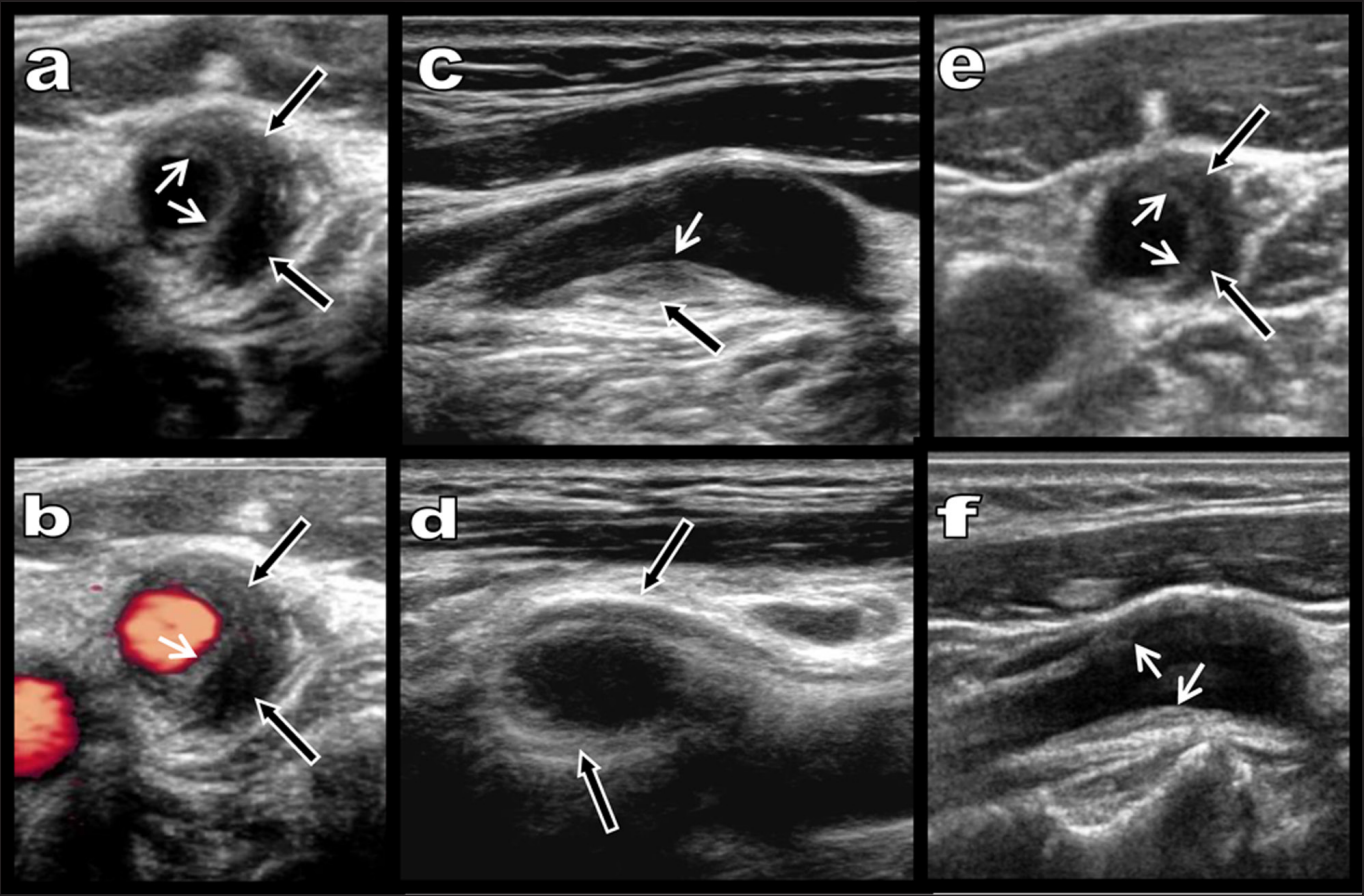
Grayscale (**a** and **c–f**) and Colour Doppler ultrasound (**b**) show a massive eccentric and preferentially laterally developed hypoechoic adventitial thickening of the bulbar internal carotid (black arrows). A thin hyperechoic intimal fibrous plaque is seen (small white arrows). Stenosis is absent. Drastic regression of the hypoechoic thickening is already found after only 15 days of treatment with non steroidal anti-inflammatory drugs (e and f). The thin hyperchoic intimal plaque persists.

**Figure 2:**
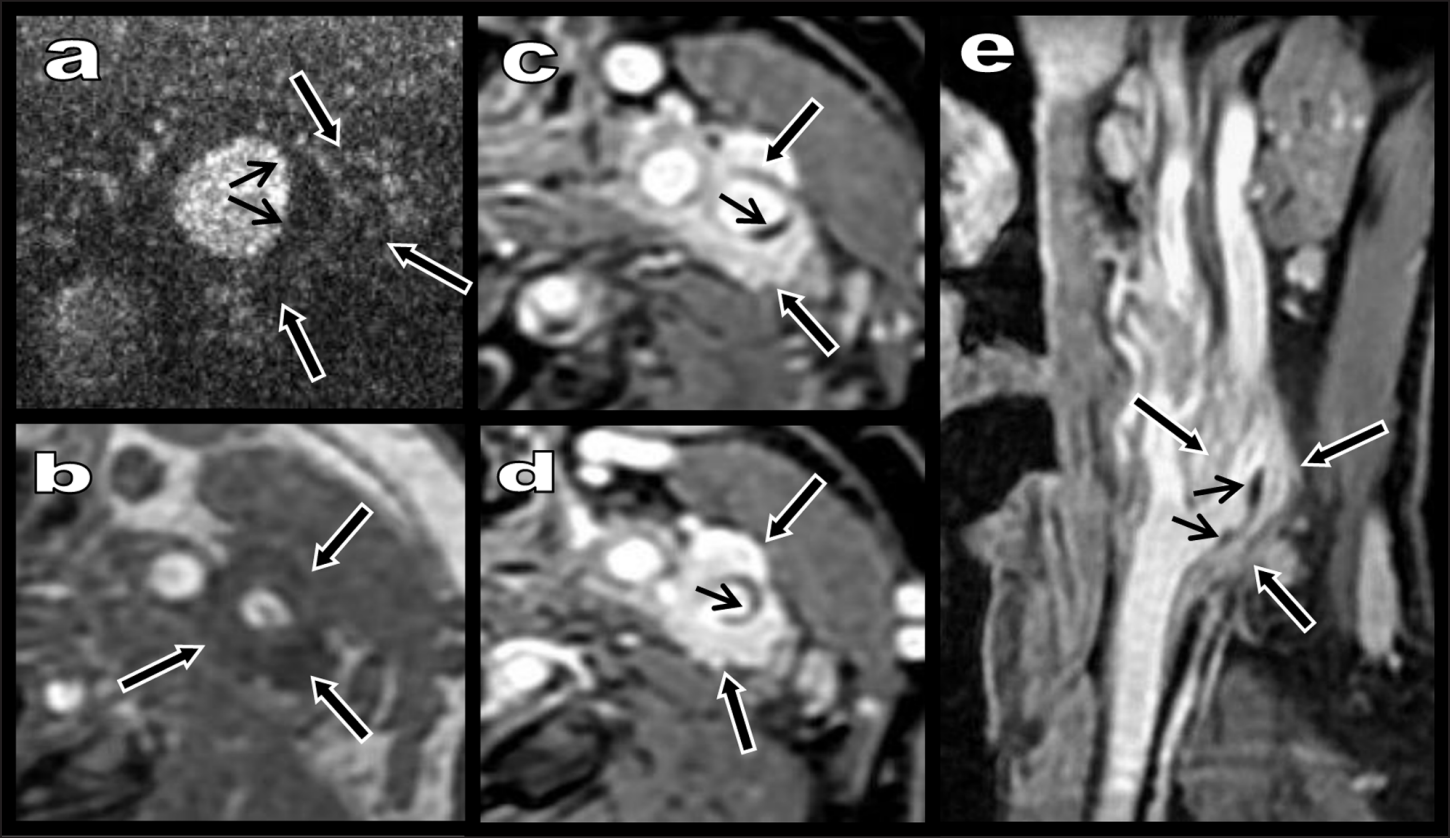
Contrast-enhanced ultrasound (a) show normal capillary distribution of micro bubbles in the hypoechoic thickening (black arrows) excluding haematoma. The avascular intimal fibrous plaque is clearly seen (small black arrows). Unenhanced Axial T1-weighted MR image (2) shows a hypointense tissue (black arrows on **a**–**e**) around the proximal internal carotid. Intense enhancement of this tissue appears on fat-saturated contrast enhanced T1-weighted images (c–e).

A 55-year-old woman reported a 24-hour history of unusual pain in her left carotid area irradiating to the ear. Colour Doppler ultrasound revealed an eccentric hypoechoic thickening (black arrows on Figure 1a–d) of the proximal bulbar internal carotid but also partially of the carotid bifurcation itself. A thin hyperechoic atheromatous fibrous plaque was also visible (small white arrows on Figure 1a–f) but no significant stenosis was found. Contrast-enhanced ultrasound showed normal capillary distribution of micro bubbles in the hypo echoic thickening, therefore excluding haematoma (Figure 2a, black arrows). The avascular fibrous plaque was well demonstrated (small black arrows). Unenhanced Axial T1-weighted Magnetic Resonance (MR) imaging showed an hypo intense tissue (Figure 2b, black arrows) around the proximal internal carotid. Intense enhancement of this tissue was shown on fat-saturated contrast enhanced T1-weighted images (Figure 2c–e). Carotidynia or TIPIC syndrome was diagnosed and the woman was immediately treated with non-steroid anti-inflammatory drugs. Doppler ultrasound performed after 14 days already showed rapid regression of both the symptoms and the perivascular inflammatory sheath (black arrows on Figure 1e–f).

## Comment

Carotidynia is a throbbing pain exacerbated by palpation in the area of the carotid bifurcation. It was first considered to be distinct from idiopathic cervical pain and introduced in the classification of cephalic disorders in 1988. Nevertheless, carotidynia was withdrawn in 2004 because of a controversy about its relative lack of specificity. It was no longer considered to be a disease but merely a common symptom of various diseases comprising carotid dissection, vasculitis, trigeminal neuralgy, thyroiditis, sialadenitis, cervical arthrosis and infectious processes. Recent literature, however, reaffirmed carotidynia as a distinct pathological entity with structural abnormalities and characteristic radiological findings. Doppler ultrasound, Computed Tomography, MR, [^18^F] fluorodeoxyglucose positron-emission tomography and hybrid associated modalities have illustrated an abnormal amorphous enhancing soft tissue surrounding the carotid bifurcation and the proximal internal carotid suggesting an inflammatory adventitial process. A recent large multi-centric study introduced the new term “TIPIC syndrome” for “Transient Perivascular Inflammation of the Carotid artery syndrome” to better characterize the entity [1].

TIPIC is rare but probably underdiagnosed and its pathogenesis remains hypothetical. An inflammatory process of unknown origin, a reaction to certain drugs, a variant of large-vessels vasculitis or a reaction being part of an autoimmune process are considered.

The most striking feature is a unilateral (and rarely bilateral) eccentric perivascular infiltration at the level of bifurcation with a median thickness diameter of 5 mm and a median length of 20 mm. Most patients have no associated carotid stenosis. Self-limited associated soft plaque may be present in half of patients and might be induced by the healing phase of the inflammatory process. Non-steroidal anti-inflammatory drugs and high doses of aspirin are the classical treatment, usually leading to a complete relief of pain in a median delay of 13 days.
